# Lebanese Healthcare System: How Will the Aftermath Look?

**DOI:** 10.7759/cureus.10270

**Published:** 2020-09-06

**Authors:** Loulwa Farha, Joseph Abi Jaoude

**Affiliations:** 1 Faculty of Medicine, American University of Beirut, Beirut, LBN

**Keywords:** lebanon, healthcare system, explosion, tertiary medical care, primary medical care

## Abstract

The recent Lebanese port explosion came as a continuation of a series of socioeconomic disasters the country has been facing during the past year. In addition, the massive impact of the coronavirus disease-19 (COVID-19) pandemic further hastened the collapse of the Lebanese healthcare system. In light of all those events, the Lebanese healthcare sector has faced major blows that will be difficult to recuperate from. In the aftermath of the Beirut port explosion, Lebanon received immense financial and medical support from the international community in a timely fashion, which secured first level care to victims of the explosion. Nevertheless, this forced Lebanon, which was considered a prominent tertiary medical hub in the Middle East, to slowly regress into an exclusive primary care provider. As such, it is crucial for local and regional stakeholders to build strong collaborations, and shape a unified vision of Lebanon’s future healthcare system.

## Editorial

On August 4, 2020 Beirut, the capital of Lebanon, witnessed one of the biggest explosions in the history of mankind [1]. Although not really a nuclear explosion, the blast was strong enough for people from around the world to question a potential nuclear nature to the event, with some going to the extent of dubbing the explosion “BeirutShima”, owing to its similarity with the tragic events of the Hiroshima bombing [2]. The explosion led to the deaths of hundreds, wounded thousands, and resulted in a colossal physical and financial burden on the country [1]. While this particular day was truly incomparable, it merely comes as a continuation of a wave of disasters the country has faced during the past year. As a matter of fact, the recent political and economic situation has driven Lebanon into one of the most serious financial breakdowns in its modern history, and has pushed a large proportion of its population below the poverty line. In addition, the massive impact of the coronavirus disease-19 (COVID-19) pandemic further compounded the effects of the Lebanese financial collapse [3]. In light of all those events, it becomes quite evident that the major blows sustained by the Lebanese healthcare system have elicited a negative chain reaction that will be difficult to recuperate from. 

From the beginning of the Lebanese economic crisis which began around a year ago, many of the small-scale hospitals and medical centers faced threats of potential bankruptcy. However, with the continuous financial strain, even the bigger tertiary medical centers had to succumb to those dire circumstances and adopt harsh measures to stay afloat [4]. The most noteworthy measures included laying-off a considerable number of hospital staff, and closing some of the hospital wards that were operating at suboptimal capacity [4]. Moreover, many highly qualified physicians and nurses opted to leave Lebanon for better opportunities abroad as a result of pay cuts that were later accompanied by a depreciation of the national currency. As the Lebanese Pound lost approximately 80% of its value, the importation of medications and medical equipment became exceptionally difficult and restricted. As such, the pressure on the healthcare system began to build up early on, sending shock waves into the foundations of what was once considered one of the top healthcare systems in the region. As the number of COVID-19 cases drastically increased around the world, Lebanon was no exception. The COVID-19 pandemic began to pick up pace in the country just weeks prior to the explosion with infections reaching the range of a couple hundred new cases registered daily [5]. Although the absolute number may seem small, the number of COVID-19 infections is considered to be relatively high after accounting for the country’s small population size (with rates reaching as high as 250 cases per million persons as of July 2020) [5]. 

Hospitals and medical centers have been suffering for months to keep up with the increase in demand for intensive care unit beds, medical equipment, and medications necessary to cater to patients with severe COVID-19 infections. It would thus come as no surprise that the events of August 4 were the final nail in the coffin of an already toppling healthcare system. The consequences of the explosion were directly evident as the number of injured people was estimated at approximately 5,000 patients [1]. Healthcare workers who have already been suffering from psychological burnout due to the COVID-19 pandemic were also among those who lost their lives or were severely injured. To make things worse, the capital was faced with yet another repercussion that served as both a direct obstacle, and will serve as a future impediment, and that is the destruction of several hospital facilities. Three of the major tertiary medical centers serving the Beirut area were severely damaged rendering them completely non-operational, and forcing them to evacuate their patients immediately. Furthermore, most of the hospitals and medical centers in Beirut and the neighboring areas were also affected, albeit to a lesser extent. Those facilities sustained damages to their infrastructure and medical equipment which forced them to operate under harsh conditions. As such, in a matter of hours, Lebanon was faced with an acute increase in demand for healthcare, only to be met by a massive and abrupt decrease in supply. Within hours, Lebanon transformed from a major tertiary medical hub, to the equivalent of a huge emergency room, providing nothing but the most basic medical services to its patients. 

In the aftermath of all this chaos, Lebanon received and is still receiving immense financial and medical support from the international community. A significant fraction of this aid came in the form of financial funding to trusted non-governmental organizations (NGOs). Other notable support was offered in the form of basic nutrition and medical supplies, such as field hospitals, medical equipment, and medications. This remarkable aid was offered in a timely fashion, and allowed the situation in Lebanon to slightly stabilize. Although far from optimal, this secured first level care to victims of the explosion, which was of prime significance in such an exceptional situation. Nevertheless, this rapid and chaotic surge in hospital admissions further increased the rate of COVID-19 infections, which posed yet another problematic issue to an already struggling sector. 

Lebanon’s healthcare system has been on the receiving end of repeated blows that have already started to change the nature and quality of its healthcare delivery. A once prominent tertiary medical hub in the Middle East is slowly turning into an exclusive primary care provider. For that reason, it is now crucial for local and regional stakeholders to start planning out the aftermath of these unfortunate events. Despite vital support from the international community, funding is currently being distributed among several entities that lack common management that is capable of assessing the gaps in the entire sector, and allocating resources so as to reestablish a coherent system. The prevailing circumstances also necessitate the immediate resumption of functions at tertiary medical care centers that offer specialized treatments, disease screening, and disease prevention. Similarly, tackling the psychological and financial stress of healthcare personnel is essential to prevent further losses of valuable human resources. Finally, given the unstable political and socioeconomic condition in the country, it becomes of utmost importance to build a solid collaboration among the key contributors who will help shape a unified vision of Lebanon’s future healthcare system. 
